# Optimizing Eco-Friendly Degradation of Polyvinyl Chloride (PVC) Plastic Using Environmental Strains of *Malassezia* Species and *Aspergillus fumigatus*

**DOI:** 10.3390/ijms242015452

**Published:** 2023-10-22

**Authors:** Heba A. El-Dash, Nehal E. Yousef, Abeer A. Aboelazm, Zuhier A. Awan, Galal Yahya, Amira M. El-Ganiny

**Affiliations:** 1 Microbiology and Immunology Department, Faculty of Pharmacy, Zagazig University, Zagazig 44519, Egypt; hebaaldash@gmail.com (H.A.E.-D.); dr.nehalyousef@gmail.com (N.E.Y.); gmetwa@bio.uni-kl.de (G.Y.); 2 Microbiology and Immunology Department, Faculty of Medicine, Benha University, Benha 13518, Egypt; drabeeraboelazm@gmail.com; 3 Department of Clinical Biochemistry, Faculty of Medicine, King Abdulaziz University, Jeddah 21589, Saudi Arabia; zawan@kau.edu.sa; 4 Department of Molecular Genetics, Faculty of Biology, Technical University of Kaiserslautern, Paul-Ehrlich Str. 24, 67663 Kaiserslautern, Germany

**Keywords:** biodegradation, PVC plastic polymer, *Malassezia* species, Aspergillus species, depolymerase

## Abstract

Worldwide, huge amounts of plastics are being introduced into the ecosystem, causing environmental pollution. Generally, plastic biodegradation in the ecosystem takes hundreds of years. Hence, the isolation of plastic-biodegrading microorganisms and finding optimum conditions for their action is crucial. The aim of the current study is to isolate plastic-biodegrading fungi and explore optimum conditions for their action. Soil samples were gathered from landfill sites; 18 isolates were able to grow on SDA. Only 10 isolates were able to the degrade polyvinyl chloride (PVC) polymer. Four isolates displayed promising depolymerase activity. Molecular identification revealed that three isolates belong to genus *Aspergillus*, and one isolate was *Malassezia* sp. Three isolates showed superior PVC-biodegrading activity (*Aspergillus*-2, *Aspergillus*-3 and *Malassezia*) using weight reduction analysis and SEM. Two *Aspergillus* strains and *Malassezia* showed optimum growth at 40 °C, while the last strain grew better at 30 °C. Two *Aspergillus* isolates grew better at pH 8–9, and the other two isolates grow better at pH 4. Maximal depolymerase activity was monitored at 50 °C, and at slightly acidic pH in most isolates, FeCl_3_ significantly enhanced depolymerase activity in two *Aspergillus* isolates. In conclusion, the isolated fungi have promising potential to degrade PVC and can contribute to the reduction of environmental pollution in eco-friendly way.

## 1. Introduction

The inception of modern plastic polymers can be traced back to the 19th century, with significant expansion occurring in the first half of the 20th century. Plastics have revolutionized various industries, owing to their commendable attributes such as durability, strength, flexibility, lightweight nature and cost-effectiveness [1]. Regrettably, the predicament of plastic waste persists due to the absence of efficient disposal methods, resulting in its widespread accumulation in the environment. This infiltration of plastics into diverse ecosystems presents an alarming ecological threat to terrestrial and marine wildlife. Furthermore, the presence of plastic waste raises concerns about potential harm to humans, animals and plant life [2].

The repercussions of plastic waste are not limited to environmental challenges alone. The incineration of plastic waste during disposal gives rise to extensive air pollution, releasing an array of toxic gases into the atmosphere [3]. In response to this escalating issue, bioremediation has surfaced as a promising, efficient and eco-friendly approach to tackle plastic waste. It predominantly harnesses naturally occurring microorganisms for this purpose [4]. Notably, various types of bacteria and fungi have demonstrated their aptitude for biodegrading different types of plastic polymers [5,6].

Among these plastic polymers, polyvinyl chloride (PVC) stands as a prominent player in the realm of plastic materials. It is utilized in the fabrication of a diverse spectrum of products, including building materials, food packaging, children’s toys and medical supplies [7]. In terms of global production demand, PVC ranks third, following polyethylene and polypropylene [8]. PVC production is substantial, constituting approximately 10% of total plastic production, and this figure is projected to increase annually by around 3.2% [9].

PVC manifests in both rigid and flexible (plasticized) forms. The plasticized variant incorporates additives that augment its flexibility and durability, rendering it a versatile material suitable for a wide range of applications. However, the extensive utilization and persistence of PVC in the environment underscore the urgent need for sustainable strategies to address its biodegradation and removal. This is crucial for alleviating environmental pollution and reducing the associated risks to ecosystems and human health. Landfill and incineration are the most widely used methods for PVC disposal [6]. Unfortunately, PVC leaches poisonous chemicals into surrounding media, causing toxicity to humans [10]. Furthermore, burning PVC releases hydrogen chloride gas and other harmful combustion products [11].

Although PVC is notoriously resistant to biodegradation, there exists a body of research indicating the potential for certain bacteria and fungi to break down this recalcitrant polymer [8,12,13]. Fungi, in particular, have emerged as promising candidates for PVC biodegradation. Fungi exhibit the remarkable capability to degrade a wide range of organic chemicals with remarkable versatility, as they lack stringent specificity requirements. Additionally, fungi can thrive across a broad spectrum of pH levels, making them adaptable biodegradation agents [14].

Moreover, fungi produce hydrophobins, a group of proteins that play a pivotal role in facilitating attachment to hydrophobic substrates. This initial step of attachment is of paramount importance in the biodegradation process [15]. Filamentous fungi are more efficient than yeast in biodegradation process, as hyphal apical growth allows fungi to penetrate into plastic materials easily, in addition to the secretion of both exoenzymes and hydrophobins [6]. Several genera of fungi play an essential role in plastic biodegradation including *Aspergillus*, *Paecilomyces*, *Fusarium*, *Geomyces*, *Nectria* and *Penicillium* [16,17,18,19].

Fungi employ various mechanisms for polymer degradation, involving both enzymatic processes and physical disruption. The pivotal plastic depolymerization process hinges on the action of certain enzymes, with notable examples being hydrolases, like cutinase, lipase and depolymerases [20]. Cutinase, for instance, is proficient in hydrolyzing high-molar-mass polyesters [21], while lipase demonstrates effectiveness in breaking down various polymer types [22]. Depolymerases, a class of hydrolases, exhibit the ability to target a range of plastic polymers [23].

Recent research indicates that a more efficient approach to plastic degradation is to employ enzymes produced through a commercial process [20]. This strategy underscores the importance of optimizing factors such as pH, temperature and plastic concentration during enzyme production. Given that microorganisms exhibit varying performance levels under different pH and temperature conditions, it is crucial to evaluate their efficacy across various pH and temperature ranges [24]. Methods employed to assess plastic degradation primarily involve determining mass loss, identifying the appearance of chemical metabolites or observing surface deterioration due to microbial growth and enzyme activity [25].

The objective of the present study is to isolate fungi capable of degrading plastics from soil contaminated with plastic waste. Subsequently, the study aims to evaluate the depolymerase enzyme activity of these fungi under different conditions, including variations in temperature, pH and the presence of diverse ions and buffers. This investigation seeks to identify the optimal conditions for the growth and plastic-degrading activity of these fungal isolates.

## 2. Results

### 2.1. Isolation and Screening of Plastic Degrading Fungi

Out of the 100 samples collected from sites with accumulated plastic waste, 18 fungal strains exhibited the ability to grow on Sabouraud dextrose agar (SDA). Further screening revealed that 10 of these isolated fungal strains were able to flourish on Bushnell Hass agar (BHA) when PVC served as the sole carbon source. Notably, only four isolates displayed the capability to generate a distinct zone of clearance on the BHA/PVC medium. (Table 1).

### 2.2. Depolymerase and Lipase Enzyme Activities (Quantitative Method)

Of the 10 isolates discussed in the previous step, only four isolates showed depolymerase activity of more than 0.01 U (0.032, 0.014, 0.032 and 0.024 U/mL/min of activity for isolates F1, F2, F3 and F4, respectively). Only two isolates showed lipase enzyme activity (isolates F1 and F4 with 0.02 and 0.017 U activity, respectively), as shown in Table 1. The four tested isolate did not have any esterase activity.

### 2.3. Phenotypic Identification, Molecular Identification and Phylogenic Analysis

**Figure 1:**
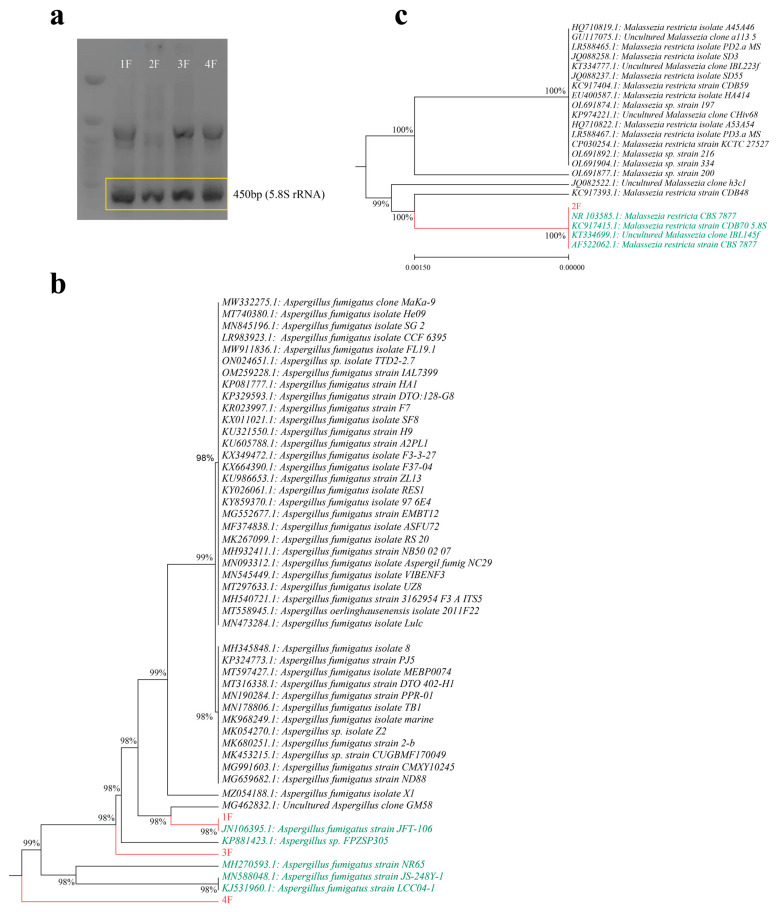
Molecular identification and phylogenetic analysis of fungal isolates. (**a**) Gel electrophoresis of 5.8S *rRNA* (amplicon size 454 bp). (**b**) Phylogenetic tree of the partial 5.8S rRNA gene sequences from isolates F1, F3 and F4 (in red) compared to sequences of the most related (in green) *Aspergillus* sp. strains recognized by BLASTN. (**c**) Phylogenetic tree of the partial 5.8S *rRNA* gene sequence from isolate F2 (in red) compared to sequences of the most related (in green) *Malassezia* sp. strains recognized by BLASTN.

The four isolates displaying the highest depolymerase activity were subjected to phenotypical identification and 5.8S rRNA sequencing. These isolates exhibited different growth characteristics on culture media, segregating them into two distinct categories based on colony shape and color. Isolates 1, 3, and 4 shared similar growth patterns, forming green colonies, while isolate 2 produced white colonies (Supplementary Figure S1). Analysis of the 5.8S rRNA amplicon through sequencing (Figure 1a) further classified these four isolates into two primary genera: *Aspergillus* sp. and *Malassezia* sp. A phylogenetic assessment, which considered the degree of homology between the 5.8S rRNA sequences of these isolates and those of closely related species in the GenBank, revealed that isolates F1, F3 and F4 were identified as *Aspergillus fumigatus*, whereas isolate F2 was categorized as *Malassezia* sp. (Figure 1b,c).

### 2.4. Hydrophobicity Assay

The hydrophobicity assay revealed that *Aspergillus*^3^ (strain F4) exhibited the highest hydrophobicity at 82.29%, followed by *Malassezia* (strain F2), *Aspergillus*^2^ (strain F3) and *Aspergillus*^1^ (strain F1) with hydrophobicity percentages of 68.82%, 58.12% and 41.86%, respectively (Table 1).

### 2.5. Weight Reduction Analysis and SEM Analysis of PVC Strips

Weight reduction analysis demonstrated that strain *A. fumigatus*^3^ exhibited the highest reduction (2.15 ± 0.42%), followed by strains *A*. *fumigatus*^2^, *Malassezia* sp. and *A*. *fumigatus*^1^, with reduction percentages of 1.92 ± 0.51, 1.46 ± 0.7 and 0.718 ± 0.1, respectively (Table 1).

**Figure 2:**
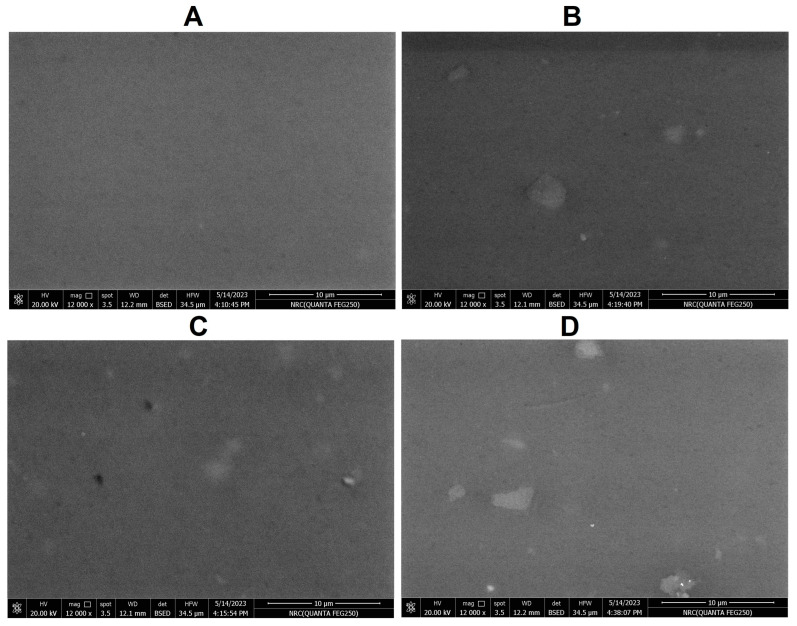
SEM micrographs showing biodegradation of PVC strips by isolated fungal strains. (**A**) control (untreated PVC strips), (**B**) PVC strips treated with *Malassezia* sp. (strain F2), (**C**) PVC strips treated with *A. fumigatus*^2^ (strain F3) and (**D**) PVC-strips treated with *A. fumigatus*^3^ (strain F4). The PVC strips treated with fungal isolates showed cracks and erosion. Scale bar is 10 µm.

SEM analysis revealed that *A*. *fumigatus*^3^, *A. fumigatus*^2^ and *Malassezia* sp. strains were capable of creating surface cracks on the PVC strips, with the most substantial erosion observed in the *A*. *fumigatus*^3^ strain. In contrast, SEM images of control PVC strips displayed no surface erosion (Figure 2).

### 2.6. Effect of pH and Temperature on Fungal Growth

The impact of varying pH on the growth rate was assessed by measuring the optical density after 24 h at pH levels ranging from 4 to 10 (as summarized in Table 2). The results indicated that strains F1 and F4 exhibited superior growth rates under alkaline conditions (pH 8–9), whereas strains F2 and F3 displayed optimal growth at acidic pH (pH 4). Similarly, the influence of temperature variation on growth was investigated after a 24 h incubation period at the optimal pH for each isolate, across a range of temperatures (30, 40, 50 and 60 °C), with the findings presented in Table 2. Notably, the optimum growth temperature was determined to be 40 °C for strains F1, F2 and F4, whereas strain F3 (*A. fumigatus*^2^) displayed more robust growth at 30 °C.

### 2.7. Depolymerase Enzyme Activity at Different Temperatures and pH

**Figure 3:**
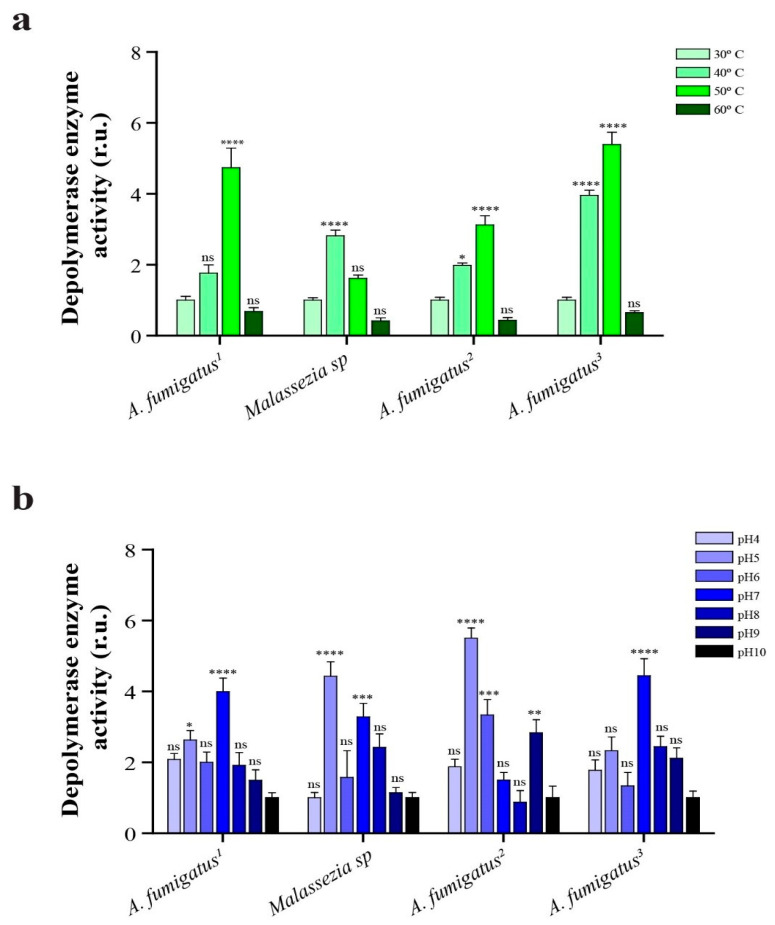
Relative depolymerase enzyme activity of *Aspergillus* and *Malassezia* strains at: (**a**) different temperatures (at pH 7) and (**b**) different pH (at temperature 37 °C). Data were normalized to 30 °C in case of (**a**) and to pH 10 in case of (**b**). r.u. (relative units), ns (no significance), * *p* < 0.05, ** *p* < 0.01, *** *p* < 0.001 and **** *p* < 0.0001.

The depolymerase enzymes produced by the *Aspergillus fumigatus* strains exhibited higher activity at 50 °C, whereas the enzyme from *Malassezia* sp. displayed its peak activity at 40 °C. Changing the temperature from the reference level of room temperature (30 °C) to 40 °C, 50 °C and 60 °C yielded statistically significant differences at all tested temperatures (*p* value < 0.05), indicating a substantial impact of temperature variation on the depolymerase enzyme activity. Figure 3a illustrates the relative depolymerase activity in comparison to the activity at 30 °C. Notably, the maximum depolymerase activity was observed at 50 °C among all the *Aspergillus* isolates, with a marked decrease when the temperature was raised to 60 °C.

Furthermore, *Aspergillus fumigatus* isolates F1 and F4 demonstrated superior activity at pH 7. In contrast, *A*. *fumigatus* isolate F3 and *Malassezia* sp. exhibited higher activity at pH 5, as depicted in Figure 3b. The depolymerase activity of all isolates was found to be optimal at slightly acidic or neutral pH conditions. Conversely, under alkaline pH conditions, depolymerase enzyme activity significantly decreased, except for *A*. *fumigatus* isolate F3, which exhibited efficient activity even at pH 9. Figure 2b illustrates the relative depolymerase activity (compared to activity at pH 10).

### 2.8. Depolymerase Enzyme Activity in Presence of Different Concentrations of EDTA

**Figure 4:**
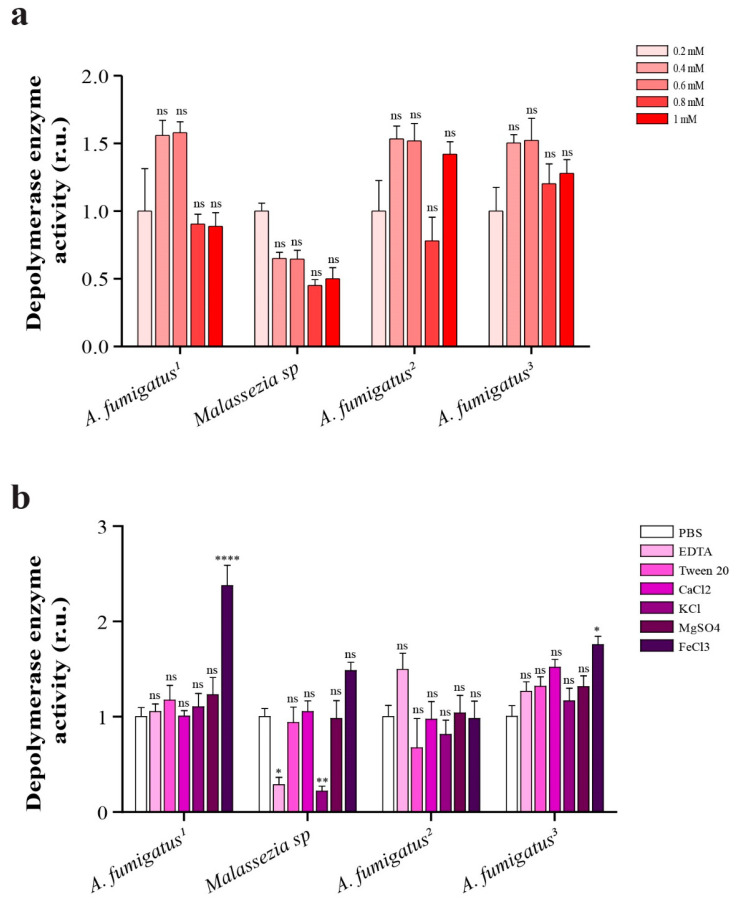
Relative depolymerase enzyme activity of *Aspergillus* and *Malassezia* strains in (**a**) presence of different concentrations of EDTA, (**b**) presence of 1 mM EDTA, Ca^2+^, K^1+^, Mg^2+^, Fe^3+^ and 1% Tween 80 or PBS. Data were normalized to 0.2 mM EDTA in case of (**a**) and to PBS in case of (**b**). r.u. (relative units), ns (no significance), * *p* < 0.05, ** *p* < 0.01 and **** *p* < 0.0001.

The effect of varying concentrations of EDTA is depicted in Figure 3a. Employing different concentrations of EDTA revealed no statistically significant differences (*p* value > 0.05) in the depolymerase activity of both the *Aspergillus* and *Malassezia* strains. Notably, the activity of the *Aspergillus* strains was markedly enhanced in the presence of EDTA compared to *Malassezia.* Figure 4a illustrates the relative depolymerase activity in comparison to the activity at 0.2 mM EDTA.

### 2.9. Comparison of Depolymerase Enzyme Activity in Presence of 1 mM (EDTA, Ca^2+^, K^1+^, Mg^2+^, Fe^3+^) and 1% v/v Tween 80

The effect of 1 mM (CaCl_2_, KCl, MgSO_4_) and 1% *v*/*v* Tween 80 showed a non-significant difference in the depolymerase activity among *Aspergillus* strains, However, the presence of FeCl_3_ significantly enhanced the depolymerase activity in the case of *Aspergillus fumigatus* (isolates F1 and F4). A remarkable reduction in the depolymerase activity was consistently detected upon treatment with 1 mM EDTA or KCl in the case of *Malassezia* sp., as shown in Figure 4b.

## 3. Discussion

Plastic polymer production has emerged in the last 150 years. Its properties, such as flexibility, durability and strength, have made it superior to glass and metals in many fields [26]. Unfortunately, the accumulation of plastic waste, due to the slow degradation of plastic polymers, has had a negative impact on the environment [27]. Plastic biodegradation has been identified as the cleanest method for addressing the issue of plastic waste. Our study aimed to search for fungi that could assist in PVC polymer biodegradation and to evaluate the effect of different temperatures, pH levels, ions and buffers on the depolymerizing enzymes secreted by the identified fungal species. Among the isolated fungi, four strains displayed promising depolymerase activity; three of them were identified as *Aspergillus fumigatus*, and the last strain was found to be *Malassezia* sp.

*Aspergillus* is a filamentous ascomycete fungus and is one of the most abundant genera on Earth, owing to its tolerance towards a wide range of environments [28]. *A. fumigatus* has been recognized as an efficient recycler in nature and is a dominant fungus in soil, constituting approximately 35–70% of the total soil fungi [29]. In the current study, three *Aspergillus fumigatus* strains were isolated from soil samples, and these isolates were able to form zone of clearance on BHA containing PVC as a sole carbon source. In addition, they showed promising depolymerase activities (0.032, 0.032 and 0.024 U/mL/min). Similarly, a previous study reported that *A*. *fumigatus* was able to grow actively on natural and synthetic polyester polymers [30]. Furthermore, soil isolates of *A*. *fumigatus* were able to degrade poly-β-hydroxybutyrate acid (PHB) biopolymers [31].

On the other hand, *Malassezia* sp. is well known as an opportunistic yeast that inhabits the skin and mucosa of humans and warm-blooded animals, leading to skin disorders and fungemia [32]. However, the occurrence of *Malassezia* sp. in diverse ecological niches has been demonstrated in genetic-based culture-independent studies. For example, DNA sequences similar to *Malassezia restricta* have been detected in diverse habitats, including deep-sea sediments [33]. In addition, *Malassezia pachydermatis* was found to be widespread in nature, and it is one of the *Malassezia* sp. that can grow on a lipid-free medium such as SDA [34,35].

In our study, *Malassezia* sp. was isolated from soil and was able to biodegrade PVC polymers. Similarly, Amend detected *Malassezia* in marine environments that contained plastic wastes [33]. In the current study, *Malassezia* sp. was able to produce depolymerase enzymes, which is novel information about *Malassezia*’s role in the environment. Li and coworkers found that *Malassezia* isolates can produce protease enzymes that have hydrolytic activity on bacterial biofilm [36]. Protease is one of the enzymes that can degrade polluting plastic polymers [37]. Altogether, this supports our results that *Malassezia* can play a role in plastic biodegradation. Our data revealed that the optimum conditions for *Malassezia* growth were 40 °C and pH 4, while the optimum conditions for depolymerase enzyme activity were 50 °C and pH 5.

Hydrophobic cells play a key role in removing contaminants from soil and water, as hydrophobic cells adhere more strongly to hydrophobic surfaces [38]. *A*. *fumigatus* conidia are considerably hydrophobic [39]. In the current study, *A*. *fumigatus* (strain F4) has the highest hydrophobicity (82.29%), followed by *Malassezia* sp., then *Aspergillus* (strain F3) and *Aspergillus* (strain F1). Concurrent with our results, a recent study reported that *Aspergillus niger* isolates were more hydrophobic than the *Aureobasidium pullulans* isolate [28], as *Aspergillus niger* strains had low absorbance in the polar phase (9.2–11.7%), meaning that they have a high affinity for the oily (hydrophobic) phase.

Although *A*. *fumigatus* grew optimally at 37 °C and a pH of 3.7–7.6, it can be isolated from soil with temperatures ranging from 12 °C to 65 °C and a pH range of 2.1–8.8 [39]. Our results showed that the optimum temperature for the growth of *A*. *fumigatus* isolates on PVC polymer is between 30 °C–40 °C, and the optimum pH for growth was variable, ranging from 4 to 9 for the three *Aspergillus* isolates. Scherer found that 30 °C is the optimum temperature for the growth of *A*. *fumigatus* [30]. While Aly and coworkers [31] reported that the maximum PHB depolymerase production by *A*. *fumigatus* was at pH 5 and 30 °C. In addition, Jung and his colleagues showed that *A*. *fumigatus* strain 76T-3 grew better at a temperature of 40 °C [23]. While Al Hosni and coworkers found that the most suitable temperature for *A*. *fumigatus* to biodegrade polycaprolactone (PCL) is 25–37 °C [40]. All of these studies support our results that showed variable growth and plastic degradation activities by different fungal strains and indicate the necessity of finding the optimum conditions of action for each strain.

PVC biodegradation involves three main steps: the chain depolymerization, oxidation and mineralization of formed intermediates [6]. In the current study, the four fungal isolates showed depolymerase activities of 0.032, 0.014, 0.032 and 0.024 U/mL/min. Furthermore, depolymerase enzymes produced by two of our *A*. *fumigatus* isolates exhibited optimal activity at pH 7 and a temperature of 50 °C, while the depolymerase of the third *Aspergillus* strain demonstrated peak efficiency at pH 5 and a temperature of 40 °C. It is worth noting that a previous study indicated that the enzymatic degradation of polyethylene terephthalate is limited to 50 °C (around 5% degradation), and degradation increases with higher temperatures (55–65 °C) to exceed 30%. However, it is important to consider that such elevated temperatures may hinder microbial growth [41]. Jung and his colleagues found that *A*. *fumigatus* strain 76T-3 exhibits optimal depolymerase enzyme activity at pH 6.5 and a temperature of 55 °C [23]. In a related study, Iyer and his team demonstrated that depolymerase enzymes tend to have their peak activity at alkaline pH levels (around 8.5) and temperatures ranging from 45 °C to 60 °C [42]. Therefore, there is a pressing need to extract thermally stable enzymes from fungal strains and employ them for plastic biodegradation under carefully selected optimal conditions.

The most widely used method for evaluating plastic biodegradation is the weight reduction analysis. Our analysis of PVC strips treated with fungal strains for 30 days revealed that *A*. *fumigatus*^3^ displayed the most substantial reduction (2.15% ± 0.42%), followed by *A*. *fumigatus*^2^, *Malassezia* sp. and *A*. *fumigatus*^1^ with reductions of 1.92% ± 0.51, 1.46% ± 0.7 and 0.718% ± 0.1, respectively. Importantly, these weight reduction results align with the findings from our hydrophobicity assay, where strains with the highest hydrophobicity induced the most significant weight loss.

In a recent study, PVC samples degraded by 10% and 32% after 1 month of incubation with *Aspergillus niger* and *Aspergillus glaucus*, respectively [43]. Additionally, weight reductions ranging from 15% to 20% were observed for PVC treated with fungal strains over a 2-month period [44]. The relatively higher weight reduction in these studies can be attributed to the use of commercially available plasticized PVC, which is more susceptible to microbial attack [45]. Similarly, a more substantial weight loss (ranging from 3.521% to 26.15%) was observed for low-density polyethylene (LDPE) when treated with a fungal consortium of *Aspergillus niger*, *Aspergillus flavus* and *Aspergillus oryzae* over 55 days [46]. The higher reduction in Dsouza’s study may be due to the use of a mixture of three fungal strains, an extended incubation period and the use of enriched growth media (PDA). In contrast, a lower weight reduction (0.3% ± 0.06%) was observed for LDPE treated with fungi recovered from landfill sites [47].

In our current study, we employed SEM to further investigate the degradation of the PVC strips. SEM micrographs revealed the presence of erosions and cracks in PVC films treated with fungi, in contrast to control untreated PVC strips. These observations are consistent with the results from several previous studies, as they can be attributed to the colonization of fungal strips with fungal strains and the formation of biofilms [43,44,45,46,47].

In our research, we also assessed the impact of using EDTA, Fe^3+^, Ca^2+^, K^1+^, Mg^2+^ and Tween 80 separately on depolymerase activity. Notably, only the addition of FeCl_3_ significantly enhanced depolymerase activity in two *Aspergillus* isolates. While the depolymerase of *Aspergillus* strains showed enhanced activity at EDTA concentrations ranging from 0.4 to 0.6 mM, statistical analysis revealed that changes in EDTA concentration had no significant effect on depolymerase enzyme activity. Similarly, the separate addition of Ca^+2^, K^+1^, Mg^+2^ and Tween 80 showed statistically insignificant changes in depolymerase enzyme activity. This aligns with previous research, which demonstrated that EDTA is able to inhibit various polymerase enzymes from different *Penicillium* sp. [48]. Furthermore, a recent study reported that the use of Tween 80 surfactant had no significant impact on improving the biodegradation rates of low-density polyethylene (LDPE) using *Aspergillus* spp. [43].

Numerous studies have reported the use of fungi, including *Aspergillus* sp., for the biodegradation of various plastic polymers [49,50,51,52,53,54]. *Aspergillus* sp. has demonstrated the capacity to biodegrade LDPE [43,49,50,51], HDPE [52,53], polyurethane [54], and Polybutylene succinate-co-adipate (PBSA) polymer [55]. Only two studies have characterized PVC degradation by *Aspergillus* sp. One study reported that *A*. *niger* and *Aspergillus sydowii* can biodegrade PVC polymer [12]. Another recent study showed that *A*. *niger* and *Aspergillus glaucus* were capable of degrading PVC polymers [43]. There is a raised concern that some of the fungal strains (including *Aspergillus* and *Malassezia* species) could release toxic metabolites or mycotoxins during their growth [56] and that these metabolites may have a negative impact on the environment (if the degradation process occurs *in situ*). This problem can be solved by performing the biodegradation process under controlled conditions (either at the laboratory scale or at higher scales) or by extracting the fungal enzymes and using them in the biodegradation process instead of using the fungal strain itself.

In conclusion, we have isolated fungal strains with promising activity for degrading PVC plastic polymers. To the best of our knowledge, our study is the first to explore the potential role of *Aspergillus fumigates* and *Malassezia* sp. in PVC plastic polymer degradation. In addition, we found that the optimal conditions for growth differ from that of enzyme activity for most of the strains. Also, these optimum conditions are different between strains of the same species. Hence, it is recommended to grow fungi first at the optimum temperature and pH for them to obtain the highest levels of depolymerase enzymes. Then, the depolymerase enzyme can be extracted and purified to be used at its optimum temperature and pH to obtain the most benefit from its use. This will help to break down plastic waste in a shorter time and help to restore balance to the environment.

Future work will concentrate on investigating the mechanisms of action of enzymes extracted from the most successful strains. Also, it is a necessity to make the enzymatic degradation of plastic a commercial process. Indeed, the environmental strain genus *Aspergillus* would be the perfect fungus for developing commercial levels of plastic biodegradation [49].

## 4. Materials and Methods

### 4.1. Polymer Preparation

The PVC polymer was an industrial-grade polymer supplied by Elreda Company (Gharbya, Egypt) in the form of powder. Polymer emulsion was prepared as described previously with some modifications [57]. Briefly, 1 g of PVC was suspended in 20 mL of dichloromethane and 30 mL of distilled water. This mixture was sonicated for 10 min. Then, dichloromethane was evaporated by stirring at 80 °C in water bath for 2 h, then the pH was adjusted to 7 with KOH. The obtained suspension was added to 1 L of culture media to obtain a final concentration of 0.1% PVC.

### 4.2. Plastic Degrading Isolates

A total of 100 soil samples were collected from different landfill locations (harboring plastic waste) in Benha, Qualibya, Egypt. Samples were collected in sterile falcon tubes and transported to a microbiology laboratory for processing. One gram of sample was transferred into a flask containing 99 mL of sterile distilled H_2_O, and the flask was shaken vigorously to suspend soil particles. The pour plate method was used for the isolation of fungal species associated with soil, using Sabouraud dextrose agar (SDA, Oxoid, Hampshire, UK) as culture media, SDA plates were incubated at 30 °C for 2–7 days. The developed colonies were picked and streaked on SDA to obtain a pure single colony for each fungal isolate that was kept on slants at 4 °C [58]. For long-term storage, spores were harvested in sterile water (by rubbing the surface of SDA plate with a sterile glass rod), spore suspension were stored in sterile 50% glycerol at −20 °C [59].

### 4.3. Qualitative Assay of Plastic Biodegradation (Zone of Clearance Method)

Bushnell Hass agar (BHA) media (consisting of the following components: MgSO_4_ 1 g, KH_2_PO_4_ 1 g, K_2_HPO_4_ 1 g, NH_4_NO_3_ 1 g, CaCl_2_ 0.02 g, FeCl_3_ 0.05 g and 20 g of Agar in 1000 mL of dH_2_O) was prepared and PVC polymer (previously prepared) was added to this media to a final concentration of 0.1%. BHA plates containing PVC as the sole carbon source were used to identify plastic degrading fungi through the formation of a clear zone around colonies. The obtained fungal isolates were inoculated onto BHA plates, which were incubated at 28 °C for up to 21 days. Clear zones surrounding the colonies were periodically observed, and the diameter of the zone of clearance was measured; isolates that had clear zones surrounding them were used in further experiments [58].

### 4.4. Fungal Spore Hydrophobicity Assay

Fungal isolates were grown on SDA at 28 °C for 4–5 days to allow fugal sporulation. Spores were harvested by flooding with 10 mL of sterile H_2_O and gently rubbing the surface using glass rod; the resulting spore suspension was collected using micropipette. The collected spore suspensions were filtered through sterile filter paper (Whatman No. 1) to remove any mycelial fragments. Spore hydrophobicity assay was performed as described previously [60]. Briefly, spore suspensions with approximately equal number were prepared using a hemocytometer, the initial number of spores defined as N_0_. Spores were suspended in 3 mL mineral oil/water mix (1:1 *v*/*v*) and shaken vigorously. After settlement, the number of spores remaining in the water (N) was counted. The percent portioning in oily phase was calculated as: (1 − N/N_0_) × 100. The results were the average of three independent experiments.

### 4.5. Quantitative Determination of Depolymerase Enzyme Activity

Isolated fungal species were inoculated in Bushnell Hass broth (BHB) supplemented with PVC as a sole carbon source and incubated for up to 21 days. The crude extracellular enzymes were collected via centrifugation at 10,000× *g* for 10 min. Then, the supernatant was filtered using 0.22 µm SCA-grade cellulose acetate membrane filter to obtain a culture-free supernatant [61].

The activity of depolymerase enzyme was measured according to the method of Kobayashi et al. (1999) with slight modifications [62]. The assay mixture contained 50 µL of BHB-containing plastic polymer, 50 µL of phosphate-buffered saline (PBS) and 100 µL of crude enzyme, which was extracted previously. The optical density (OD) was measured every 10 min. for 60 min using MB-580-Microplate Elisa Reader, BIORAD, (Heales, Shanghai Chemdo Co., Ltd., Shanghai, China). One unit of depolymerase activity was defined as a 0.001 OD decrease in absorbance at 650 nm per min. Wells containing only 100 µL of BHB containing PVC and 100 µL PBS were used as control. Each experiment was repeated 3 times, and the average was calculated for each strain [63].

### 4.6. Quantitative Determination of Esterase and Lipase Enzyme Activities

The assay was performed according to the protocol of Ramnath et al. (2017) with some modifications [64]. Briefly, the reaction mixture contains 50 µL PBS, 50 µL p-nitrophenol (p-NP) acetate ester dissolved in methanol and 100 µL of crude enzyme extract (extracted previously). Absorbance was measured at 405 nm, at the beginning of the reaction and after being incubated for 30 min at 30 °C. One unit of esterase activity is defined as the amount of enzyme producing 1 mol of p-NP per minute. Control wells contained only 50 µL p-NP ester and 100 µL of PBS. Each experiment was repeated 3 times, and the average was calculated.

The reaction mixture contained 100 µL of pre-warmed (50 °C) 1% tributyrin emulsion in PBS and 100 µL of crude enzyme extract. The reaction was monitored at room temperature (21 to 23 °C) by measuring the OD of the emulsion at 450 nm for 5 min every 30 s. Control wells contained only 100 µL 1% tributyrin emulsion in PBS. Each experiment was repeated 3 times, and the average was calculated [61].

### 4.7. Molecular Identification of Isolates via 5.8S-rRNA Sequencing and Phylogenetic Analysis

The genomic DNA (gDNA) was extracted from the four isolates that showed highest depolymerase activity, as described previously with slight modifications [65]. Briefly, 50 µL of spore suspension (~10^7^ spores/mL) was prepared from 3D-old colonies in sterile water then heated to 100 °C for 10 min using a Biometra T-GRADIENT thermocycler (Rudolf-Wissell-Str. Göttingen, Germany). The spore suspension was centrifuged at 16,000× *g* for 5 min to pellet debris. A total of 1 µL of supernatant was used as template DNA in the next step.

Polymerase chain reaction (PCR) was performed in a Biometra thermocycler. Primers used for the amplification of fungal 5.8S-rRNA were supplied from Sigma Aldrich (Petaluma, CA, USA). These primers are the universal primers ITS86-F (**5**′-GTGAATCATCGAATCTTTGAA-**3**′) and ITS4-R (**5**′-TCCTCCGCTTATTGATATGC-**3′**) and have been described previously [66]. COSMO PCR RED 2x Master Mix (Willowfort UK, Birmingham, UK) was used for amplification. Amplification mixture was prepared in a volume of 20 μL and consists of: 10 µL of Master Mix, 1 µL of each of forward and reverse primers, 2 μL of DNA template and 6 µL of nuclease-free water. Cycling conditions include initial denaturation at 94 °C for 3 min followed by 35 cycles of denaturation at 45 s, annealing for 45 s at variable temperature (55 °C for isolate 1, 3 and 4 and 59 °C for isolate 2) and extension at 72 °C for 1 min. A final extension cycle at 72 °C for 10 min was used [67,68].

PCR products was detected using agarose gel electrophoresis according to Sambrook et al. [69]. PCR products were purified using Thermo Scientific GeneJET PCR purification kits (Vilnius, Lithuania) following the manufacturer’s instructions. The purified PCR products were used for sequencing, where 5 μL of template DNA (20–80 ng) were mixed with 5 μL of ITS86-F primer (5 pmol/μL). PCR samples were sequenced using the Illumina HiSeq platform using 300 PE chemistry (GATC-Biotech, Konstanz, Germany, part of Eurofins Genomics Germany GmbH). The 5.8S rRNA sequences were used to build the phylogenetic trees using MEGA X software [70]. The 5.8S *rRNA* sequences identified in this study have been deposited in the GenBank and were given accession numbers, as indicated in the Data Availability Statement.

### 4.8. Analysis of Plastic Biodegradation via the Weight Reduction Method and Scanning Electron Microscope (SEM)

Colorless PVC plastic strips (Polysolutions Co., Ltd., Changhui Shanghai, China) were cut into small pieces (10 × 2 cm) and weighed accurately (W1). Each piece was placed separately in a tube containing BHB media and then autoclaved for 20 min. Each fungal isolate was inoculated in triplicates and incubated for 1 month at 37 °C. After incubation, the PVC strips were washed with sterile dH_2_O and dried, then weighed again (W2). Weight reduction was calculated using the following equation: (W1 − W2)/W1 × 100. Average weight reduction (for triplicate measurements) was calculated for each isolate [58].

For the assessment of structural changes of PVC strips, the surface morphology of PVC films (with ≥1% weight reduction from previous experiment) was analyzed using SEM (model QUANTA FEG 250, Thermo-Fisher Scientific, New York, NY, USA). PVC strips (from previous experiments) were completely washed with sterile dH_2_O, then strips were gold-coated using S150A Sputter Coater (Edwards company, Midlands, UK). The images of PVC strips treated with fungi were compared with those of the control, comprising untreated PVC strips [43].

### 4.9. Effect of pH and Temperature on Fungal Growth

Isolated fungal species were grown in Sabouraud dextrose broth (SDB) for 24 h at 30 °C, then 1 mL of media was transferred to 9 mL of BHB containing PVC polymer prepared at different pH, ranging from 4 to 10, changing by one degree increase each time, and the pH of BHB/PVC medium was adjusted using drops of concentrated NaOH (to obtain alkaline pH) or HCl (to obtain acidic pH). The cultured tubes were incubated for 48 h at 37 °C. The absorbance was measured at 620 nm after 24 h and after 48 h to detect the best pH for fungal growth (by comparing OD at 24 h with that at time-point 0 of inoculation) [71]. The previous steps were repeated using different temperatures (30 °C, 40 °C, 50 °C and 60 °C) for incubating BNB/PVC media inoculated with fungal strains (using the optimum pH for growth of each isolate).

### 4.10. Effect of Temperature and pH on Depolymerase Enzyme Activity

Depolymerase enzyme activity was measured on BHB containing PVC polymer using the same method described previously [63]. The procedure was performed at different temperatures (30 °C, 40 °C, 50 °C and 60 °C), by heating all the components separately in water bath then mixing them when they are heated to the required temperature then the absorbance was measured at 650 nm every 2 min for 10 min.

Furthermore, the same method was used to evaluate the effect of different pH (4, 5, 6, 7, 8, 9 and 10) on depolymerase enzyme activity. Phosphate-citrate buffer was used for preparing solutions with pH 4 (0.55 g Na_2_HPO_4_ and 0.65g Citric acid in 50 mL d H_2_O) and pH5 (0.73 g Na_2_HPO_4_, and 0.51 g Citric acid in 50 mL d H_2_O), KH_2_PO_4_/NaOH buffer for preparing pH 6 (50 mL of 0.2 M KH_2_PO_4_ and 5.6 mL of 0.2 M NaOH), pH 7 (50 mL of 0.2 M KH_2_PO_4_ and 29.1 mL of 0.2 M NaOH) and pH 8 (50 mL of 0.2 M KH_2_PO_4_ and 46.1 mL of 0.2 M NaOH). Finally, alkaline borate buffer was used for preparing solutions with pH 9 and pH 10 (50 mL of 0.2 M boric acid + 0.2 M KCl and 20.8 mL of 0.2 M NaOH). The final pH for each solution was adjusted using HCl for acidic solutions and NaOH for alkaline solutions. The assay mixture contained 50 µL of BHB containing PVC, 50 µL of previously prepared buffer solution and 100 µL of crude enzyme extract. Absorbance was measured at 650 nm every 2 min for 10 min [63].

### 4.11. Effect of Different Concentrations of EDTA, Different Ions and Tween 80 on Depolymerase Enzyme Activity

Depolymerase enzyme activity was measured in presence of different concentrations (0.2, 0.4, 0.6, 0.8, 1 mM) of Ethylene diamine tetra-acetic acid (EDTA) by replacing PBS with EDTA solution. Furthermore, depolymerase enzyme activity was measured in the presence of 1 mM of different ions (Ca^2+^, K^1+^, Mg^2+^, Fe^3+^) instead of PBS. The same experiment was carried out using 1% (*v*/*v*) Tween 80 instead of PBS [72].

### 4.12. Statistical Analysis

Data in graphs represent the means of at least 3 independent experiments ± standard errors of means (SEM) using GraphPad prism 5.0 software (GraphPad Software, San Diego, CA, USA). For statistical analysis, a two-way analysis of variance (ANOVA) test was performed, followed by Bonferroni’s post hoc test to compare replicate means [73].

## Figures and Tables

**Table 1:** Parameters of plastic degrading activity (of the isolated fungal strains) evaluated in the following study.

Isolate No.	Zone of Clearance (cm) on BHA	Depolymerase Activity (U/mL/min)	Lipase Activity (U/mL/min)	Percentage of Hydrophobicity	Weight Loss of PVC Strips
F1	4	0.032	0.02	41.86%	0.718 ± 0.1
F2	3.5	0.014	0	68.82%	1.46 ± 0.7
F3	4	0.032	0	58.12%	1.92 ± 0.51
F4	3.2	0.024	0.017	82.29%	2.15 ± 0.42%

**Table 2:** Effects of different pH and temperatures on the growth of identified fungal strains.

	*A. fumigatus* ^1^	*Malassezia* sp.	*A. fumigatus* ^2^	*A. fumigatus* ^3^
OD620 nm after 24 h incubation at different pH				
pH 4	1.11 (0.035) ^a^	5.84 (0.181) ^a^	2.35 (0.068) ^a^	1.76 (0.197) ^a^
pH 5	0.28 (0.097) ^ns^	1.76 (0.041) ^a^	1.62 (0.036) ^a^	2.33 (0.042) ^a^
pH 6	1.23 (0.034) ^a^	3.58 (0.102) ^a^	1.64 (0.055) ^a^	2.04 (0.058) ^a^
pH 7	0.67 (0.081) ^b^	0.55 (0.044) ^c^	1.40 (0.045) ^a^	3.42 (0.169) ^a^
pH 8	3.48 (0.282) ^a^	1.66 (0.032) ^a^	1.79 (0.036) ^a^	3.56 (0.053) ^a^
pH 9	2.13 (0.025) ^a^	1.83 (0.046) ^a^	1.61 (0.030) ^a^	5.08 (0.070) ^a^
pH 10 *	0.12 (0.014)	0.11 (0.032)	0.17 (0.043)	0.11 (0.023)
OD620 nm after 24 h incubation at different temperatures (optimum pH for each isolate)				
30 °C	1.05 (0.012) ^a^	1.06 (0.013) ^a^	2.87 (0.044) ^a^	1.37 (0.031) ^a^
40 °C	2.74 (0.073) ^a^	5.89 (0.058) ^a^	2.08 (0.109) ^a^	4.75 (0.066) ^a^
50 °C	0.08 (0.014) ^ns^	0.27 (0.048) ^ns^	0.34 (0.047) ^ns^	0.20 (0.033) ^ns^
60 °C *	0.14 (0.022)	0.17 (0.022)	0.31 (0.035)	0.17 (0.025)

Data represented as mean (SEM). ^ns^ (no significance), ^a^ *p* < 0.0001, ^b^ *p* < 0.01 and ^c^ *p* < 0.05. * Refers to the pH or the temperature to which other means are compared.

## Data Availability

The sequence data for strains used in this study are deposited in GeneBank, the accession numbers of these partial 5.8S-rRNA sequences are OP002278, OP002279, OP002280 and OP002281 for strains 1F, 2F, 3F and 4F, respectively.

## Supplementary Materials

The following supporting information can be downloaded at: https://www.mdpi.com/article/10.3390/ijms242015452/s1.
