# Supplementary material for: Optimizing Eco-Friendly Degradation of Polyvinyl Chloride (PVC) Plastic Using Environmental Strains of Malassezia Species and Aspergillus fumigatus
Source: Int J Mol Sci. 2023 Oct 22;24(20):15452. doi: 10.3390/ijms242015452 (PMC10607177; doi:10.3390/ijms242015452)
Supplement: Supplementary file 1 [file ijms-24-15452-s001.zip › ijms-2640668-supplementary.pdf]

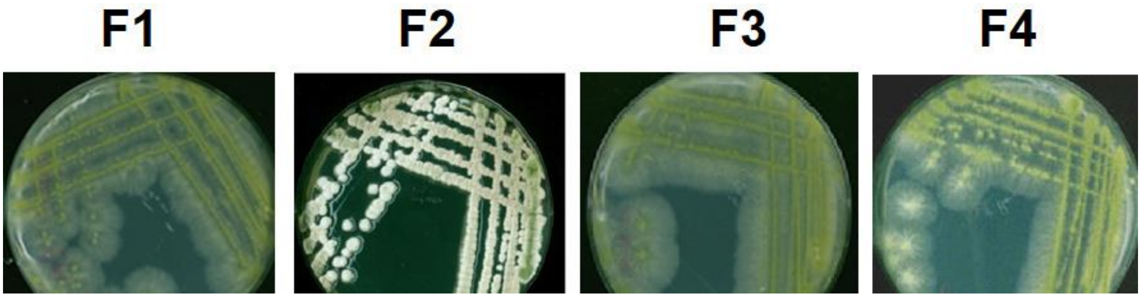

Supplementary Figure S1: Growth on SDA culture media. *Malassezia sp.* (strain F2) grew as white colonies, while *A. fumigatus*[1] (strain F1), *A. fumigatus*[2] (strain F3), and *A. fumigatus*[3] (strain F4) grew as green colonies
